# FT-Raman Spectroscopy as a Tool to Study the Secondary Structures of Wheat Gliadin Proteins

**DOI:** 10.3390/molecules26175388

**Published:** 2021-09-04

**Authors:** Iwona Stawoska, Aleksandra Wesełucha-Birczyńska, Andrzej Skoczowski, Michał Dziurka, Jacek Waga

**Affiliations:** 1 Institute of Biology, Pedagogical University of Krakow, Podchorążych 2, 30-084 Krakow, Poland; andrzej.skoczowski@up.krakow.pl; 2 Faculty of Chemistry, Jagiellonian University, Gronostajowa 2, 30-387 Kraków, Poland; birczyns@chemia.uj.edu.pl; 3 The Franciszek Górski Institute of Plant Physiology, Polish Academy of Sciences, Niezapominajek 21, 30-239 Krakow, Poland; michal.dziurka@gmail.com; 4 Department of Physiology, Plant Breeding and Seed Science, University of Agriculture, Podłużna 3, 30-239 Kraków, Poland

**Keywords:** gluten proteins, gliadins, amide I, secondary structure, Raman spectroscopy

## Abstract

Raman spectroscopy is a useful method in biological, biomedical, food, and agricultural studies, allowing the simultaneous examination of various chemical compounds and evaluation of molecular changes occurring in tested objects. The purpose of our research was to explain how the elimination of ω-fractions from the wheat gliadin complex influences the secondary structures of the remaining αβγ-gliadins. To this aim, we analyzed the endosperm of wheat kernels as well as gliadin proteins extracted from two winter wheat genotypes: wasko.gl+ (control genotype containing the full set of gliadins) and wasko.gl− (modified genotype lacking all ω-gliadins). Based on the decomposition of the amide I band, we observed a moderate increase in β-forms (sheets and turns) at the expense of α-helical and random coil structures for gliadins isolated from the flour of the wasko.gl− line. Since ω-gliadins contain no cysteine residues, they do not participate in the formation of the disulfide bridges that stabilize the protein structure. However, they can interact with other proteins via weak, low-energetic hydrogen bonds. We conclude that the elimination of ω-fractions from the gliadin complex causes minor modifications in secondary structures of the remaining gliadin proteins. In our opinion, these small, structural changes of proteins may lead to alterations in gliadin allergenicity.

## 1. Introduction

Over the past decades, Raman spectroscopy has been effectively introduced to various areas of scientific research, among others: plant biology, biomedical research as well as food and agricultural studies [1,2,3,4,5,6]. This technique allows exploring the molecular structures of biochemical compounds and the composition of the sample in a non-destructive manner [7,8,9]. To obtain Raman spectrum, one can use either dispersion or Fourier transform (FT) techniques. They differ in the wavelength, i.e., the energy of the incident radiation and the method of detection. The Fourier transform spectrometer used in our studies is equipped with lasers operating in the near-infrared range (1064 nm). This excitation line of relatively low energy is perfect for the study of plant materials and the macromolecules that build them [10,11]. In the Raman spectrum, each compound gives its characteristic bands, representing vibrational sensitivity to chemical changes, so that they can be used in qualitative analysis. Using this method, one can also perform a quantitative analysis based on the dependence of the intensity ratio of the signal intensity to the marker bands in the analyzed sample. Thanks to the above properties, it is possible to test photosynthetic dyes, polysaccharides, or nucleic acids with this spectroscopic method. One of the important applications of Raman spectroscopy is the analysis of protein secondary structures, which gives a scope to decipher the information encoded in these biomolecules [12]. Among the peptide bonds, the amide I and III bands give rise to prominent Raman bands that are correlated with the structural properties of protein molecules [12,13,14]. However, the amide III band intensity is rather low and is overlapped by other constituents present in the sample; the peak width and the asymmetry of the amide I band indicate a contribution and distribution of the proteins’ secondary structures. It is assumed that areas of the individual component peaks (obtained on the basis of decomposition of amide I band and curve-fitting analysis) correspond to their conformational contributions. The analysis of the components of the amide I band is based on the already recognized criteria as well as the fact that the generation of a reasonable fit may require additional bands (i.e., typical for aromatic amino acid side-chain vibrations) [15,16,17,18,19,20,21,22,23]. The method is applicable to determine the secondary structure of proteins in any physical state, usually liquid, which makes them inaccessible by other techniques. The analysis has been used since the 1980s and has been verified by a very good correlation with the X-ray data. In the presented studies, we have used Fourier Transform Raman spectroscopy (FT-Raman) for the investigation of wheat gluten proteins, which are commonly regarded as allergenic factors able to invoke a range of allergic symptoms in people suffering from gluten intolerance. According to Sapone et al. (2013) [24], the most commonly manifestations of gluten intolerance are as follows:

1. Celiac disease—natural gluten intolerance connected with the Human Leukocyte Antigen (HLA) system.
2. Gluten enteropathy—consequences of inflammation reaction in a small intestine.
3. Food allergy to gluten—IgE-mediated disorders of skin and alimentary tract. Induce symptoms of rhinitis, asthma, and Wheat-Dependent Exercise Induced Anaphylaxis (WDEIA).
4. Dermatitis herpetriformis—skin manifestation of gluten intolerance revealed as the skin bladder.
5. Gluten ataxia of hands, legs, and vision disorders.
6. Non-celiac gluten intolerance (e.g., the contact allergic reaction caused by gluten present in drugs, toothpastes, and cosmetics).

A serious, social problem of gluten intolerance is strongly increased by the fact that gluten occurs in a huge majority of foodstuffs (especially in baked products) and, as a consequence, plays an essential role in human nutrition and the food industry.

All of the above-mentioned variants of gluten immunological activity are strongly connected with the chemical composition and structures of gluten proteins that contain two highly polymorphic and heterogenic groups of proteins: gliadins and glutenins [25]. Their nomenclatures are based on the electrophoretic mobility of individual subunits and fractions in Acid-Polyacrylamide Gel Electrophoresis (A-PAGE) and in Sodium Dodecylsulfate Gel Electrophoresis (SDS-PAGE). Gliadins are usually divided into α, β, γ, ω-5, and ω-1.2 fractions where α are the fastest and ω-1.2 are the slowest-moving fractions during the run. It is worth emphasizing that ω-5 gliadins are the main allergens in wheat-dependent exercise-induced anaphylaxis (WDEIA)—the most dangerous, life-threatening form of wheat allergy. In turn, glutenins can be divided into high molecular weight (HMW) and low molecular weight (LMW) subunits [26]. The SDS-PAGE electropherograms are divided into four groups: A—HMW glutenins, D—D-type LMW glutenin subunits and ω-gliadins, B—B-type LMW glutenin subunits and γ-gliadins, C—C-type LMW glutenin subunits and α/β-gliadins. The slight confusion in the arrangement of protein groups in alphabetical order (A, D, B, C in spite of A, B, C, D) results from the fact that minor bands of gliadins and LMW glutenins localized in the D zone of the electropherogram were, historically, discovered later than the main proteins in zones A, B, and C [27,28].

Three amino acids—proline, glutamine and cysteine—are especially important regarding the chemical components of gluten proteins. The content of proline and glutamine in gliadins and glutenins is highly elevated in relation to other amino acid residues (about 12% and 40% of the total amino acids content, respectively). Therefore, these proteins are called prolamins [29]. On the other hand, cysteines present in both prolamin groups connect with each other via highly energetic disulfide bonds (SS), which play an important role in the stabilization of protein higher-order structures. Gliadins are able to form mainly intramolecular, while glutenins form mainly intermolecular disulfide bonds [30,31]. However, there is one exception—both subgroups of ω-gliadins are completely devoid of cysteine residues [32]. Therefore, gliadins are monomeric, while glutenins form large polymeric complexes [33]. Hence, SS play a crucial role in protein folding and the formation of stable, higher-ordered structures, influencing the allergenic properties of gluten proteins.

Another factor determining the allergenic properties of gluten proteins is the presence of the specific, short amino acid sequences in gliadin and glutenin polypeptides. These sequences play the role of epitopes, which are recognized by specific IgE class antibodies in the sera of wheat allergic persons. The attaching of IgE by epitopes initiates the immunological response of human organisms and leads to the development of disease symptoms. Pentapeptides are approved as the shortest sequences recognized by the human immunological system [34]. In gluten proteins, epitopes are usually present in regions of repetitive sequences localized close to the N-terminus in α-, β-, and γ-gliadins and LMW glutenins and in a long, central region of ω-gliadins and HMW glutenins. The good example of IgE-binding epitopes are VQQQQFPG and VRVPVPQLQP in α-gliadins, PQQPFPQQPQ and LSQQPQQTFP in γ-gliadins, or QQQLPQQQ and QQIPQQQ in ω5-gliadins, which are recognized by all WDEIA patients [35,36,37].

In order to reduce the risks associated with widespread gluten exposure, numerous research groups have undertaken studies to reduce or eliminate the allergenic properties of wheat gluten, which was widely presented by Rustgi et al. (2019) [38]. Extensive studies are oriented into two directions: technological modifications of wheat flour, mainly the enzymatic digestion of immunogenic peptides and genetic modification of wheat to eliminate or detoxicate the most allergenic gluten protein subunits and fractions [39,40]. Our research on hypoallergenic wheat resulted in the development of a number of modified genotypes that we called wasko.gl−. They lack of all ω-gliadins, some γ-gliadin fractions, and some LMW glutenins subunits [41]. On the other hand, we also developed wasko.gl+ wheat containing the full set of α, β, γ, and ω-gliadins, and it was used in our research as a control rather than a modified genotype. It is worth emphasizing that the obtained unique wheat genotypes were developed without genetic engineering but only using traditional plant breeding methods, i.e., cross combinations and selection aided by the electrophoretic markers. Furthermore, using immunochemical methods (ELISA and immunoblotting), we have evidenced that the allergenic potential of wheat gliadins in wasko.gl− significantly decreased as compared to that of wasko.gl+ [42]. In the present studies, these specific wheat lines were used as a source of closely related groups of gliadin proteins and excellent plant materials for comparative studies on gliadin secondary structures (αβγω vs. αβγ gliadins). This knowledge is still scanty (in contrast to the knowledge about primary structures and epitope sequences of gluten proteins mentioned earlier). We hypothesized that the elimination of ω-fractions from the α, β, γ, and ω-gliadin complex influences and modifies the secondary structures of the remaining α, β, and γ-gliadins. In such a case, we could expect that the decreased level of proteins’ allergenicity in wasko.gl− (observed in our previous studies) was caused not only by the elimination of allergenic ω-gliadins but also by changes in the secondary structures of the remaining proteins. Verification of this hypothesis, using the FT-Raman spectroscopic method, was the aim of the present work.

## 2. Results

**Figure 1:**
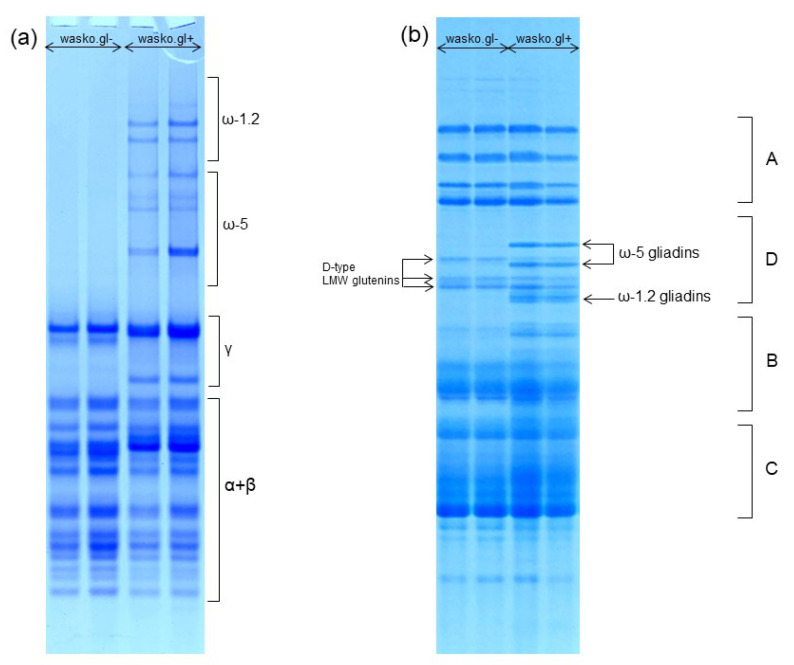
Electrophoretic patterns of wheat kernel proteins identified in wasko.gl− and wasko.gl+ wheat genotypes. (**a**): A-PAGE of gliadin proteins. The subgroups of α-, β-, γ-, ω-1.2, and ω-5 gliadins are marked on the right-hand side. (**b**): SDS-PAGE of total wheat kernel protein complex. A: HMW glutenins, D: D-type LMW glutenins and ω-gliadins, B: B-type LMW glutenins and γ-gliadins, C: C-type LMW glutenins and α/β-gliadins. Gliadins belonging to ω-1.2 and ω-5 subgroups are marked on the right-hand side, while D-type LMW glutenin subunits are marked on the left-hand side of the electropherogram.

The composition of wheat gliadin proteins and the protein complex of the whole wheat kernel were analyzed by A-PAGE and SDS-PAGE, as shown in Figure 1a,b, respectively. A-PAGE shows a complete lack of slowly migrating ω-gliadin protein bands in wasko.gl− as compared to the wasko.gl+ control line. Results obtained by A-PAGE were confirmed by electrophoresis in SDS-PAGE conditions where both ω-1.2 and ω-5 protein groups are represented by the individual protein bands. They are localized in the D-zone of the electropherogram and occur together with D-type LMW glutenin subunits (see Figure 1b). However, in contrast to A-PAGE, in SDS-PAGE, ω-1.2 gliadins migrate faster (because of their lower molecular weight) than ω-5 gliadins (of higher molecular weight). The obtained separation shows a lack of both ω-1.2 and ω-5 protein bands in wasko.gl− as compared to wasko.gl+. The three D-type LMW subunits present in wasko.gl− are used by us as biochemical markers of three null alleles responsible for the lack of ω-gliadins expression observed in this wheat genotype.

Further comparison of the gliadin composition of the kernels of the wasko.gl+ and wasko.gl− lines was performed using the high-performance liquid chromatography method.

**Figure 2:**
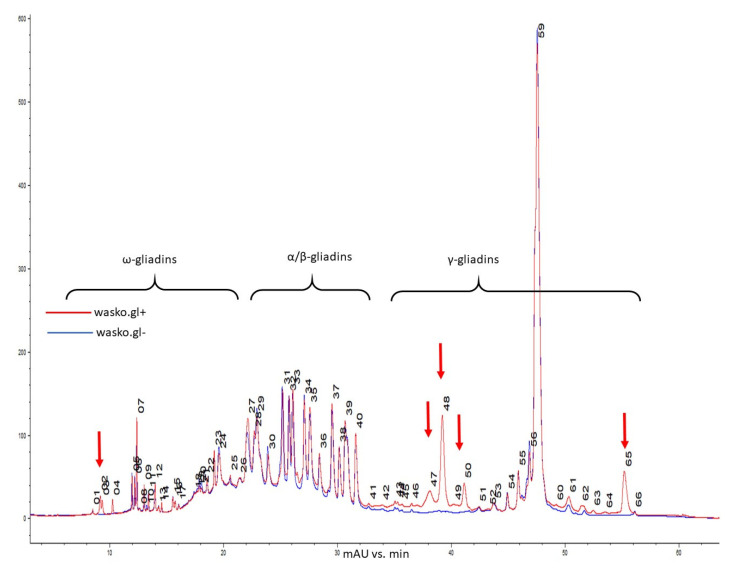
Reverse-phase high-performance liquid chromatography (RP-HPLC) comparison of gliadins composition in kernels of wasko.gl+ and wasko.gl− wheat lines, red and blue traces, respectively. Individual gliadin bands are enumerated. Arrows mark regions with missing bands in wasko.gl−.

Gliadins of wasko.gl+ and wasko.glwere separated based on hydrophobicity differences between ω (lowest hydrophobicity), α/β (medium hydrophobicity), and γ (highest hydrophobicity) fractions [41,43,44,45]. Figure 2 presents a reverse-phase chromatogram of the gliadin fraction from kernels of both lines. Comparing the wasko.gl− and wasko.gl+ lines, chromatographic analyses revealed that 12 bands decayed, of which four were assigned to ω and eight were assigned to the γ fraction. Sixteen bands decreased: seven of ω, two of α/β, and six of γ fraction. Intensities increased for 19 bands, corresponding with ω gliadins (nine peaks), α/β gliadins (seven peaks), and γ gliadins (three peaks). Still, 17 bands did not change their intensities: ω gliadins (10 peaks), α/β gliadins (two peaks), and γ gliadins (five peaks). However, comparing the total content of gliadin, which was measured as peak area, the total ω and α/β fraction did not change significantly between wasko.gl+ and wasko.gl−. However, the total content of γ gliadins in wasko.gl− decreased by one-third compared to wasko.gl+.

**Figure 3:**
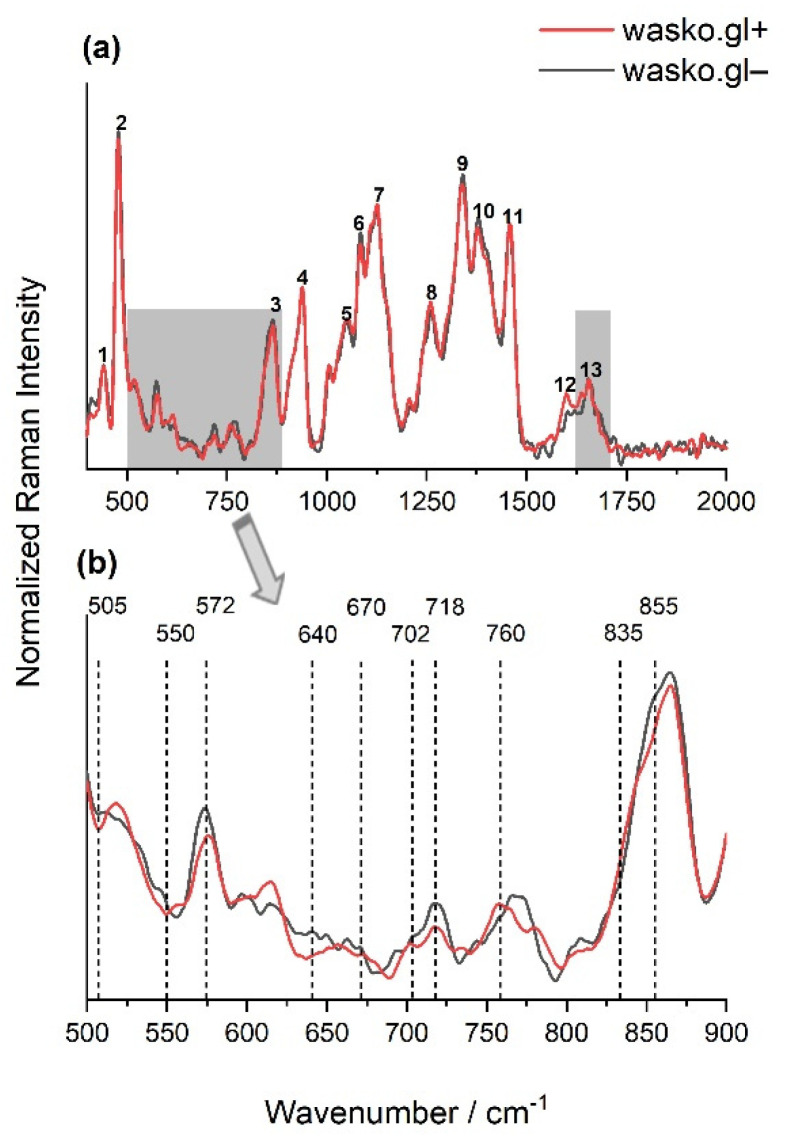
The normalized FT-Raman spectra of wheat kernels of endosperms of two lines: wasko.gl+ (red solid line) and wasko.gl− (black solid line). The spectra were measured just after cutting the kernels in half. (**a**): numbers 1–13 indicate Raman bands present in the endosperm, collected with their assignment in Table 1, with the marked 900–500 cm^−1^ and 1590–1710 cm^−1^ regions of spectra. (**b**): the region of spectra (**a**), typical for disulfide bridges conformations and aromatic acids vibrations; N ≥ 3, SD < 5%.

Fourier transform Raman Spectroscopy allowed the biochemical characterization of tested kernels of two wheat lines: wasko.gl− and wasko.gl+, without and with ω-gliadin fractions, respectively. The obtained signals were collected from the endosperm, which is the largest part of the grain, and it determines the utility value of the grains. The main component of wheat seed’s endosperm is starch (up to 80% of the total mass of the seed), which consists of two polymers: amylopectin and amylose, whereas storage proteins, compacted to the protein body and deposited in the protein storage vacuole system or the lumen of the endoplasmic reticulum [46], usually constitute up to 10–15% of the total mass. The other components such as fatty acids and fiber represent usually less than 5%. Figure 3a shows the typical Raman spectra, with the well-marked for the above-mentioned characteristic chemical groups recorded from the endosperm of wheat kernels belonging to the wasko.gl− and wasko.gl+ lines. The observed significant Raman bands and their assignments are summarized in Table 1.

The intensive Raman bands observed at 480 cm^−1^ and 940 cm^−1^ allow starch identification in various samples [48,49,50,51,52,53,54]. According to published data, the latter one is mainly due to the amylose *α*-1,4 glycosidic linkage, and changes in its intensity and position allow for the study of amylose and amylopectin contents in various samples [2,48,54]. Typical vibrations (related to C-C and C-O stretching and C-O-H deformation modes due to the glycosidic bond) observed in the region 800–1200 cm^−1^ are often called a “fingerprint” for carbohydrates [52,60,61]. Moreover, also the region between 1200 and 1500 cm^−1^ is rich in structural information about carbohydrates, thus being characteristic, among others, for C-H, CH_2_, and C-O-H deformation vibrations at 1460 cm^−1^, and coupling of the C-C-H and C-O-H deformation modes at about 1380 cm^−1^ [54,62]. The band with a maximum intensity at 1660 cm^−1^ is typical for amide I and can be used for characterization of the protein secondary structures in the sample. The peptide groups in proteins give rise also to the amide III band positioned at ca. 1260 cm^−1^, however, its intensity is rather low and overlapped by other components of measured samples.

Comparing the spectra obtained for endosperms of the tested wheat kernels of wasko.gl− and wasko.gl+ lines, the greatest differences were observed in the 500–900 cm^−1^ area and also in the 1590–1710 cm^−1^ range of amide I; see Figure 3a (gray selections). Vibrations in the 500–550 cm^−1^ region are typical for bands associated with disulfide stretching modes, which are representative mainly of sulfur-rich glutenins; see Figure 3b. According to Li-Chan (1996), S-S stretching vibrations at ca. 510 cm^−1^ are typical for gauche–gauche-gauche (SS_g-g-g_), at ca. 525 cm^−1^ for trans-gauche-gauche (SS_t-g-g_), and at ca. 545 cm^−1^ for trans-gauche-trans (SS_t-g-t_) conformations [63]. Table 2 shows the content of selected conformations, expressed as a percentage, that were obtained for the decomposition of the 500–550 cm^−1^ region measured for wheat kernels endosperms of wasko.gl− and wasko.gl+ lines. Decomposed spectra are illustrated in Figure S1 in the Supplementary Material section. As it is seen from the obtained results, the highest content of disulfide bridges was observed for SS_t-g-t_ at the expense of SS_t-g-g_ and SS_g-g-g_ conformations for kernels of the wasko.gl− line (lacking of *ω*-gliadin fractions) comparing to results calculated for kernels of the wasko.gl+ line (control wheat line with full set of gliadin proteins).

Moreover, the bands at 640–670 cm^−1^ and in the 700–745 cm^−1^ regions were assigned to the C-S stretching vibrations of methionine or/and cysteine residues. For both wheat lines, the bands at 670, 702 (sh), and 718 cm^−1^ were detected, but the peak at 640 cm^−1^, which is responsible for C-S stretching vibrations in gauche conformation [63], was only seen for kernels of wasko.gl−. It is also noteworthy that in almost all of the discussed regions, the intensity of bands for kernels of wasko.gl+ was lower compared to those of wasko.gl−; see Figure 3b. However, while disulfide bridges are the main bonds playing an important role in gluten network formation, the role of non-covalent tyrosine H-bonds cannot be omitted. The peaks at 855 and 835 cm^−1^ were assigned to tyrosine (Tyr) residues, as shown in Figure 3b, with the I(855)/I(835) ratio being used as an indicator of the tyrosine involvement in inter- and intramolecular interactions. According to Siamwiza et al. (1975), the tyrosine doublet ratio belonging to the range of 0.9–1.43 suggests that the mentioned moieties can act as both donors and acceptors of protons in a hydrogen bond [64]. However, if the ratio is higher than the upper pointed value, one can expect Tyr to act as a positive charge acceptor in the hydrogen bond. For both tested wheat lines, the tyrosine doublet was higher than 1.5; however, for the wasko.gl− line, an increase in the ratio up to 2.53 was observed, which seems to indicate the exposure of the Tyr residues and their presence in the anionic -O^−^ form. For the wasko.gl+ line, the intensity ratio I(855)/I(835) was equal to 1.84 and was close to the value obtained by Nawrocka et al. (2015) and Ferrer et al. (2011) for pure gluten samples [65,66].

Another indicator of the strength of H-bonds is the vibration position of tryptophan (Trp) residues, which are detected at 760 cm^−1^ [67,68]. Comparing the results obtained for the endosperms of both kernels, a decrease in the intensity of the Raman bands was observed from 0.14 to 0.12 for the wasko.gl+ and wasko.gl− wheat lines, respectively.

**Figure 4:**
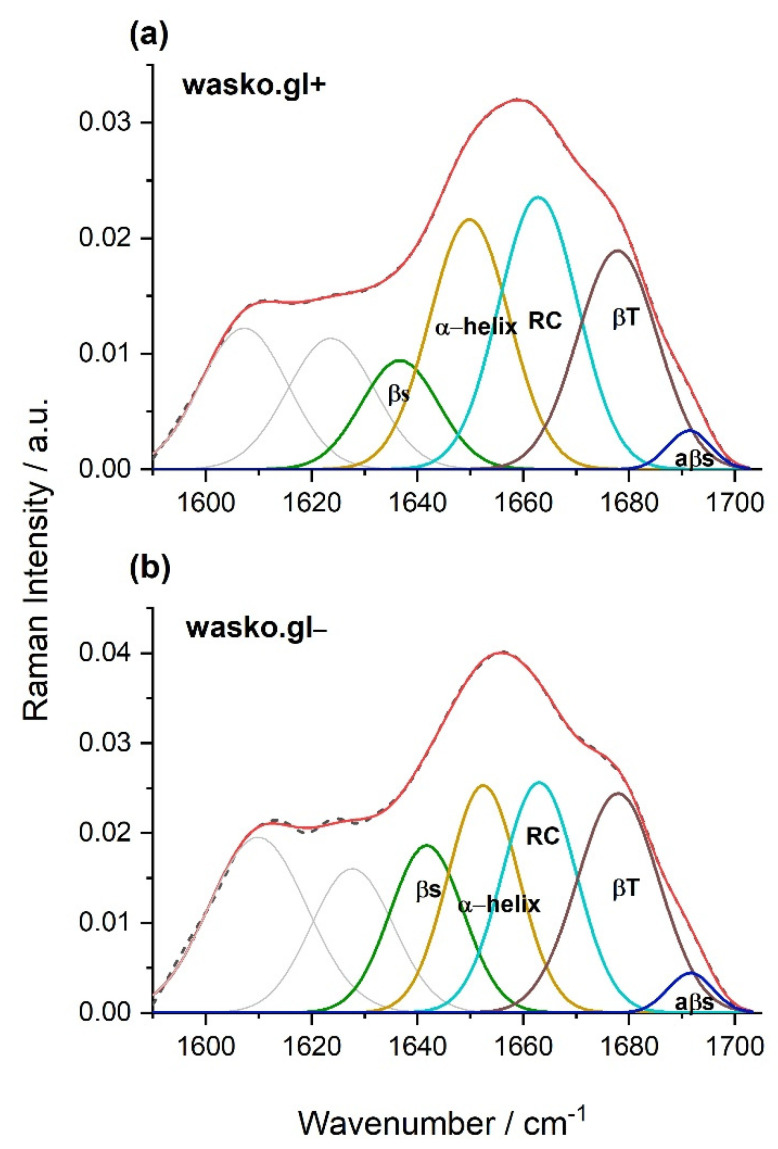
Curve fitting of the Raman spectrum in the range of amide I obtained for gliadin proteins isolated from kernels of the wheat lines wasko.gl+, panel (**a**), and wasko.gl−, panel (**b**). The experimental spectra are represented by dashed black lines and the calculated ones are represented by solid red lines. The calculated profiles in the panels were determined as the sums of the individual curve-fitted components, namely, β-sheet (1634–1640 cm^−1^—green), α-helical (1649–1652 cm^−1^—yellow), random coil (1663 cm^−1^—magenta), β-turn (1677–1678 cm^−1^—brown), and aβ-sheet (1690–1692 cm^−1^—dark blue) structures.

As indicated earlier, comparing the spectra obtained for the endosperm of the tested kernels of the wasko.gl− and wasko.gl+ lines, the greatest differences were observed not only in the range of disulfide bridges vibrations or the aromatic acids region but also in the range of amide I: 1590–1710 cm^−1^. Both tested wheat lines differed from each other in the fractions of ω-gliadin proteins; see Figure 1. Therefore, we conclude that the observed changes in the amide I region registered for kernels, as shown in Figure 3, are the result of differences in the composition of the gliadin proteins, which in turn may affect their secondary structures. To prove this suggestion, we isolated the gliadin proteins from the flour, made the Raman spectra of proteins from the liquid samples, and finally decomposed the amide I bands. As a result of the performed decomposition, four components were obtained, which allowed for the identification of various types of secondary structures [15,16,17]; see Table 3 and Figure 4. Furthermore, an additional two bands positioned in the ranges 1607–1610 cm^−1^ and 1620–1627 cm^−1^ were identified as the aromatic amino acid side chine vibrations, which are characteristic for phenylalanine (Phe), tryptophan (Trp), and tyrosine (Tyr) residues [18,19,20,21,22,23].

The β-sheet structures are visible at the frequencies 1634–1640 and 1690–1692 cm^−1^ with the latter being typical for structures rich in intermolecular hydrogen bonds (antiparalell β-sheets, aβ-sheet). The β-turn fraction is detected at 1677–1678 cm^−1^, and α-helical structures are found in the range of 1649–1652 cm^−1^. Additionally, the band localized at 1663 cm^−1^ is connected to a random coil (undefined structures). Based on the percentage of gliadin proteins secondary structures, it can be concluded that for the wheat line wasko.gl−, a higher content of β-structures (sheets and turns) was observed at the expense of α-helix and disordered coils or undefined structures.

**Figure 5:**
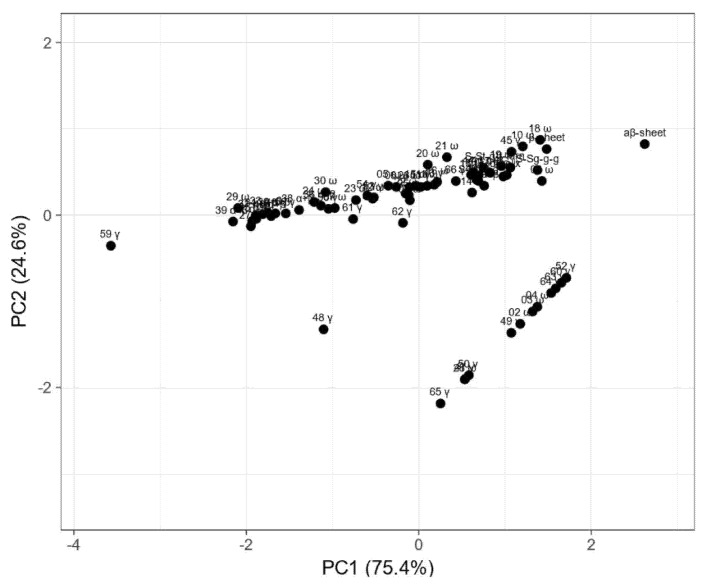
Principal component analysis (PCA) plot of measured proportions of gliadin fraction and secondary structure parameters estimated from Raman spectra. Values are ln(x)-transformed. Unit variance scaling is applied to rows; singular value decomposition (SVD) with imputation is used to calculate principal components. Principal component 1 (PC1) explains 75.4% and principal component 2 (PC2) explains 24.6% of the total variance.

Principal component analysis (PCA) was done to visualize the relationship between changes of the gliadin fraction composition and secondary structure elements estimated based on the Raman spectra of wasko.gl+ and wasko.gl− lines, as shown in Figure 5.

**Figure 6:**
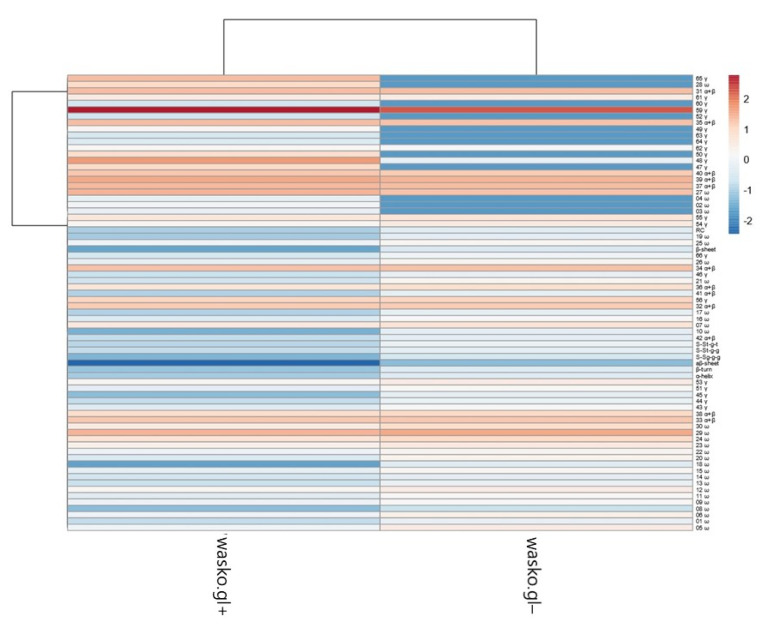
Heat map of analyzed gliadin fractions and secondary structure parameters. Original values are ln(x)-transformed. Unit variance scaling is applied to columns. Both rows and columns are clustered using correlation distance and average linkage. Coloring intensity represents the relative value of a particular parameter. Red shades represent accumulation, whereas blue shades represent parameter decrease.

The PCA plot presents two principal components, PC1 and PC2, which respectively explain 75.4% and 24.6% of the total variance. The first principal component increases with parameters concerning secondary structure such as the β-sheet, β-turn, α-helical, random coil, and aβ-sheet structures and sulfur bonds. The strongest positive correlation is observed with aβ-sheet structures. A negative correlation occurs for most medium hydrophobic gliadin fractions, i.e., α/β. The strongest negative correlation is observed with the γ-gliadin 59th band, which accumulates in both seed lines at the same level. The second PC correlates positively with all secondary structure parameters and low- and medium hydrophobic gliadin factions (ω and α/β), whereas negative correlation occurs with γ-gliadins. The strongest correlation occurs for γ-gliadin bands disappearing in wasko.gl−. This data matrix is visualized in Figure 6 as a heat map. The coloring gives an overview of numeric differences. A separation among ω and γ decaying in wasko.gl− and the rest of the gliadins (common for both lines) together with secondary structure parameters is visible. This suggests that the Raman properties of both lines regarding the protein fraction are mainly determined by low and medium hydrophobic gluten fractions, i.e., ω- and α/β-gliadins.

## 3. Discussion

We analyzed gliadin proteins extracted from two, closely related winter wheat genotypes: wasko.gl+ (containing the full set of gliadin protein fractions) and wasko.gl− (containing the same set of gliadins but lacking of all ω-gliadins, as shown in Figure 1). The goal of our studies was to explain whether and how the elimination of ω-fractions from the complex of αβγω-gliadins influences the secondary structures of the remaining αβγ-gliadins. The FT-Raman method allows us to study the differentiation of secondary structures in both groups of proteins (αβγω and αβγ gliadins). As mentioned earlier, ω-gliadins lack cysteine residues; hence, they cannot join other gluten proteins via SS bonds. They are proteins of the lowest hydrophobicity among all gliadins, which is greatly illustrated by the chromatograms of RP-HPLC, where individual protein fractions are separated according to their increasing surface hydrophobicity. In this method, ω-gliadins elute earliest, at the very beginning of the analysis, which confirms their rather hydrophilic character. Contrarily, αβγ-gliadins contain cysteine residues, which are able to create secondary structures stabilized by intramolecular SS bonds. Furthermore, they are highly hydrophobic and may aggregate with other gluten proteins via hydrophobic interactions. In the gluten protein complex, ω-gliadins can interact with other proteins via weak, low energetic hydrogen bonds. Therefore, we could expect that the elimination of ω-fractions from the gliadin complex may cause only minor modifications in the secondary structures of the remaining αβγ-gliadins. Based on the decomposition of the amide I band calculated for liquid samples of gliadins, we observed a decreasing of α-helix and random coils and an increasing in aggregated forms and β-structures (both sheets and turns) in gliadins without ω-fractions.

The presented studies enabled us to verify the hypothesis that the elimination of ω-gliadins (from the wheat kernels, obtained for the wasko.gl− line that was proved previously as less allergenic comparing to the wasko.gl+ line [42]) influences the gluten structure and the secondary structure of the gliadin complex.

FT-Raman spectroscopic measurements were carried out on both (i) kernel endosperm and (ii) gliadin fractions isolated from flour obtained from tested wasko.gl− and wasko.gl+ wheat lines. The first stage of the measurements allowed us to characterize the vibrations typical of biological macrobiomolecules present in the tested samples, and it confirmed our assumption that the elimination of the ω-gliadin fractions can influence, to some extent, conformational changes in protein structures. The SS_g-g-g_ conformation, which is typical of the most stable protein structures, was detected in the higher content in the endosperms of the wasko.gl+ line compared to the wasko.gl−. Interestingly, the highest content of SS_t-g-t_ structures with relatively low stability of disulfide bridges were found in the wasko.gl− kernels, as shown in Table 1. A similar result, but with a different percentage of each type of SS conformation, was observed by Nawrocka et al. [65,69]. These authors examined the changes in the conformation of pure gluten SS bridges after fiber modifications, and they found that the addition of dietary fiber to pure gluten samples led to a decrease in the SS_g-g-g_ conformation with an increase in the SS_t-g-g_ and SS_t-g-t_ structures. On the basis of the obtained results, it can be concluded that the lower content of gliadin proteins in the endosperm of wheat kernels of wasko.gl−, precisely the lack of ω-gliadin fraction, leads to the transformation of SS bridges from SS_g-g-g_ and SS_t-g-g_ to SS_t-g-t_ structures of lower SS bond stability. It is also possible that the higher content of SS_t-g-t_ and also SS_t-g-g_ structures observed in the kernels of the wasko.gl− line may be connected to changes in protein folding and possibly also with the aggregation of gluten proteins [70], which confirms the results of amide I decomposition obtained for gliadin proteins (fraction of gluten protein) extracted from kernels (the second stage of the Raman studies), as shown in Table 3 and Figure 4. For the gliadins extracted from the wasko.gl− line, we observed a lower content of α-helix and RC forms compared to wasko.gl+, which is a result of structural changes in the above-mentioned protein complex. It is worth emphasizing that the band observed at 1620–1627 cm^−1^ is also classified in the literature as the one that is typical for aggregated structures that may arise as a result of intermolecular hydrogen-bonded β-sheets [65,71]. For gliadins extracted from kernels of the wasko.gl− line, the maximum of this band was found at a higher frequency (1627 cm^−1^) than for gliadins obtained from kernels of the wasko.gl+ line (1624 cm^−1^). Thus, it is likely that not only aromatic amino acid side-chain vibration but also the increasing amounts of beta-sheets, which may contribute to the formation of aggregated structures, are a part of this band and contribute to its shift toward higher frequencies.

Tyrosines can be formed in the repeats of a doublet of tyrosine residues present in gluten proteins. The ratio of the intensity of the tyrosine peaks at 855 and 835 allow us to discuss the contribution of these moieties in inter- and intramolecular interactions. The value of the intensity ratio of I(855)/I(835) obtained for wasko.gl− suggests that the phenyl groups are rather weakly bound by the H- bond (even partial exposure of the Tyr phenyl group may take place), and the wasko.gl+ gluten proteins were more closely matched compared to those of the wasko.gl− line. The obtained results are in line with the literature data presented by Ferrer et al. and Nawrocka et al. [66,69], in which the authors claimed that the protein folding may be hindered by the exposition of Tyr moieties detected by the increased value of the doublet of tyrosine.

FT-Raman spectra also show the band associated with the Trp oscillations, which were due to the indole ring vibration (at 760 cm^−1^). The observed changes in the intensity of this band suggest that Trp residues of the wasko.gl− line (the line devoid of ω-gliadin fractions) are exposed on the surface of the protein complex and may play a role in the formation of a more disordered structure [68].

As pointed out above, the decomposition of the amide I band of gliadin proteins into component structures indicates the higher content of β-sheet and β-turn structures for kernels for the wasko.gl− line compared to the wasko.gl+ line. This indicates that Trp residues were locally involved in the formation of hydrogen bonds and as a consequence may lead to the aggregation of gluten proteins (especially gliadin).

Interestingly, the results of our analyses indicate that secondary structure parameters, derived from Raman data, correlate with moderately hydrophobic gliadin fractions (higher hydrophobic γ, all α/β, and ω-gliadins of lower hydrophobicity), as shown in Figure 5. This leads us to suppose that these gluten fractions are mainly responsible for the final properties of wheat flour. Taking all of that into account, and on the basis of reports of other researchers [72,73,74,75], it can be hypothesized that the reduction of allergenicity, during the classic process of breading assisted by electrophoretic phenotyping, was achieved potentially without a negative impact on the technological properties of flour. To a certain extent, it can be noticed in Figure 6, where both lines’ differences are flattened when considering most of the gluten structures (secondary structure parameters, medium hydrophobic gliadin fractions). The main differences are connected only with low hydrophobic ω- and γ-gliadins.

We can conclude that the structural changes in the analyzed gliadin proteins, which are related to the lack of ω-gliadin fractions in the wasko.gl− wheat line, may be one of the reasons for the changes in the gliadins’ allergenicity observed by us earlier [41]. In our previous studies on gliadins’ allergenicity analyzed by immunoblotting [42], we observed that native gliadins do not show any binding with IgE antibodies from the sera of wheat allergic individuals. Instead, after the reduction of gliadins with mercaptoethanol, we observed a strong signal of chemiluminescence for all immunoreactive protein bands. This fact allows us to conclude that gliadins after the reduction of SS bonds may expose hidden epitopes from the inside to the outer surface of the protein molecule, facilitating their IgE binding.

The FT-Raman analysis showed that the elimination of ω-gliadins from the gliadin complex may cause some changes resulting in the relaxation and partial unfolding of αβγ-gliadin structures, which may favor the initiation of immunological response. It is worth emphasizing that the observed changes also applied to SS bonds. These results indicate that changes in the secondary structures of gliadins after elimination of the ω-fractions may—as in our previous studies—facilitate the exposure of IgE epitopes to interaction with IgE antibodies.

## 4. Materials and Methods

### 4.1. Wheat Lines

Kernel samples of wasko.gl+ and wasko.gl− winter wheat genotypes used as plant material for FTR analysis in these studies were obtained from individual wheat plants reproduced on experimental plots in the Department of Physiology, Plant Breeding and Seed Science, University of Agriculture in Kraków, and harvested in 2020. Both kernel halves and wheat flour were the objects of our investigation.

### 4.2. Sample Preparation

Samples of whole grains were preliminarily prepared by grinding in a mortar and next used for HPLC (Agilent Technologies, Woldbron, Germany) and electrophoresis analysis (Hoefer, Holliston, MA, USA) as well as gliadins’ extraction prior to FT-Raman (Thermo Scientific Nicolet NXR 9650 FT-Raman spectrometer, Waltham, MA, USA) measurement. For FT-Raman analysis of biochemical components, whole grains were cut into half and used for spectroscopic measurements.

### 4.3. Gliadins Extraction

In order to extract gliadins from flour, first, water-soluble proteins were separated. To do this, 0.15 mol/dm^3^ NaCl (1:2 *v*/*w*) was added into the solid sample and gentle shaken for 2 h at room temperature. After centrifugation (13,000 rpm, 10 min), the extract was removed, and the pellet was dried using inert gas (nitrogen). Next, gliadins were extracted with 70% EtOH (1:1.5 *v*/*w*) and shaking overnight at room temperature. Finally, the sample was centrifuged (13,000 rpm, 10 min), and the supernatant was used for further measurements.

### 4.4. Acid Polyacrylamide Gel Electrophoresis (A-PAGE) of Wheat Gliadins

Gliadin proteins were extracted from flour of wasko.gl− and wasko.gl+ wheat lines, using 70% ethanol, overnight. Before analysis, gliadin extracts were concentrated with saturated sucrose solution in the aluminum lactate buffer (pH 3.1). Proteins were separated by native A-PAGE in aluminum lactate buffer, pH = 3.1, according to the methods worked out by Bushuk and Zillman (1978) [76] and Metakovsky and Novoselkaya (1991) [77], with several modifications. Gliadin extracts were introduced into wells in gel slabs of total monomer concentration T = 8% (*w*/*v*) and the crosslinker (methylene bisacrylamide) concentration C = 0.29% (*w*/*v*). Electrophoresis was done at constant voltage (U = 500 V) and current (I = 80 mA) in the aluminum lactate buffer, pH 3.1, in a Hoefer SV400 electrophoretic chamber (Hoefer, Holliston, MA, USA) for about 4 h. Gels were stained in Coomassie Brilliant Blue (0.026%), methanol (17%), and trichloroacetic acid (5%) solution overnight and then de-stained in distilled water. De-stained gliadin gels were recorded using a Lumix FZ 1000 digital camera and archived as JPEG files.

### 4.5. Sodium Dodecyl Sulfate Polyacrylamide Gel Electrophoresis (SDS-PAGE) of Wheat Total Proteins

The complex of total wheat proteins was extracted from the flour of wasko.gl− and wasko.gl+ wheat lines, using a sample buffer composed on 6 M urea, 2.5% SDS, and 1.5% mercaptoethanol with the addition of a pinch of bromophenol blue, overnight. Alkaline SDS-PAGE was performed using a standard method developed by Laemmli with several modifications as described earlier [78,79,80]. Shortly, the obtained protein extracts, composed on HMW and LMW glutenins, gliadins, albumins, and globulins, were loaded on the gel slab of dimension 18 × 16 cm in two repetitions. Proteins were separated on the polyacrylamide gel of total monomers concentration T = 10% and crosslinker concentration C = 1.23% in Tris-HCl buffer (pH = 8.1) using a Hoefer SE 400 electrophoretic apparatus (Hoefer, Holliston, MA, USA). Separation was carried out for 4 h at a constant current I = 90 mA, at 4 °C. Voltage values varied during the run from 80 to 500 V. For the visualization of all separated proteins, gels were stained after electrophoresis in Coomassie Brilliant Blue R 250 (0.02%) and G 250 (0.006%) solution overnight and de-stained in water for one day. As in gliadins, de-stained total protein gels were recorded using a Lumix FZ 1000 digital camera and archived as JPEG files. The classification of separated wheat proteins into A, D, B, and C groups was according to Shewry et al. (1999) [26].

### 4.6. Reverse-Phase High-Performance Liquid Chromatography (RP-HPLC) of Gliadin Fraction

Changes in gliadins proportions were estimated using RP-HPLC [41,43]. Seed material was milled in the MM400 mixing mill (Retsch, Kroll, Germany) using stainless steel adapters and beads. Samples of 100 mg were extracted 70% ethanol for 1 h at ambient temperature in a 2 mL polypropylene screw vial in MM400. Then, samples were centrifuged (13,000 rpm, 5 min, Universal-R, Hettich, Haan, Germany), and the clear supernatant was analyzed by RP-HPLC. Analyses were done on binary system Agilent Infinity 1260 (Agilent Technologies, Woldbron, Germany) with a DAD detector. Separation was achieved on an AdvanceBio RP-mAb SB-C8 (SB-C8) 4,6 × 150 mm with fused-core 3.5 µm particles (Agilent Technologies, Santa Clara, CA, USA). Gradient separation was used, H_2_O (A) and acetonitrile (B), with 0.1% trifluoroacetic acid (TFA) in both phases. The separation started at 25% B, then in 13 min, B increased to 45%, then in 65 min, B reached 50%. The injection was 20 µL, and the flow rate was 0.8 mL/min at 60 °C. Absorbance was recorded at 210 nm.

### 4.7. Statistical Analysis

Singular value decomposition (SVD) with imputation was used to calculate principal components. Original values were ln(x)-transformed. PCA and a heat map was done with the use of an online tool (http://biit.cs.ut.ee/clustvis/ accessed: 25 July 2021) according Metsalu and Vilo (2015) [81].

### 4.8. FT-Raman Spectroscopy and Curve Fitting

All spectra were performed with the Thermo Scientific Nicolet NXR 9650 FT-Raman spectrometer (USA) equipped with a Micro-Stage Microscope and the InGaAs (indium gallium arsenide) detector. The samples were excited with a 1064 nm line of the Nd:YAG3+ laser. FT-Raman measurements were performed for (i) the air-dried wheat kernels of wasko.gl+ and wasko.gl− lines, which were cut into half just before the experiment, and for (ii) the liquid samples of gliadins isolated from flour obtained from the above-mentioned wheat lines. The spectra were collected in the range of 4000–300 cm^−1^, accumulated from 500 and 2000 scans for solid and liquid samples, respectively, and measured with the laser power of 0.5 W for kernels (it was monitored if it did not injure the endosperm—the studied part of the grains) or 0.8 W for liquids. Each presented result is an average of at least 3 independent spectra. Spectra obtained for kernels were analyzed in the range of 2000–300 cm^−1^ starting from the baseline correction in order to extract Raman signals from the registered results containing the fluorescence background contribution originating from the intrinsic fluorescence of the kernels’ molecules. Striving to elaborate a reliable procedure for quantitative analysis of the obtained results, the spectra were normalized to the most typical band present in each one at 1460 cm^−1^, which was related to C-H vibrations originating from the deformation vibrations of the CH_3_, CH_2_, and CH functional groups in lipids, amino acid side chains of the proteins, and carbohydrates. The mathematical analysis was carried out using OriginPro 2020 software packages for Windows. To determine the percentage distribution of the disulfide bridges conformation of gluten proteins present in the endosperm of kernels, decomposition of the bands localized in a range of 500–550 cm^−1^ was done. Determination of the secondary structures of gliadin proteins extracted from wheat kernels of wasko.gl+ and wasko.gl− lines was obtained based on a decomposition of the amide I (1590–1710 cm^−1^) band registered for liquid samples. For the mathematical analysis, namely decompositions calculations, the PeakFit 4.12 (Systat Software, Inc., Chicago, IL, USA) program was used according to the modified procedure described earlier [2,12]. The examination started with a baseline correction that used a linear function. In the next step, a second derivative of each measured spectrum was obtained to find the number of components that built a selected band. Finally, we used a mathematical algorithm, employing Gaussian and Lorentzian functions, to iteratively estimate parameters using the method of least squares. In this method, the areas of selected peaks correspond to their conformational contributions. The iteration procedure, which ensures the matching of individual components to the experimental spectrum, stops when it achieves the best fit—one that cannot be improved anymore. The analysis of the conformations of SS bridges, as well as components of the amide I band, is based on the already recognized criteria as well as the fact that the generation of a reasonable fit may require additional bands (i.e., those typical for aggregated protein forms); in this wavenumber range, there are also marker bands from certain aromatic amino acids [15,16,17,18,19,20,21,22,23,63]. It is assumed that areas of the individual component peaks (obtained on the basis of decomposition of the SS-bridges region and amide I band and curve-fitting analysis) correspond to their conformational contributions. Regarding disulfide bridges conformation and the secondary structures of gliadins estimated from amide I decomposition, the correlation coefficient was not lower than 0.9985 and 0.9991, respectively.

## Figures and Tables

**Table 1:** The most characteristic Raman bands obtained for the wheat kernels of wasko.gl+ and wasko.gl− lines.

Peak Number	Wavenumber/cm^−1^	Chemical Components	References
1	440	polysaccharides (skeletal mode of pyranose ring vibrations)	[47,48]
2	480	polysaccharides (marker band to identify presence of starch)	[48,49,50,51,52,53]
3	865	mono-, di- and polysaccharides (stretching or deformation modes referring to the glycosidic bond)	[49,50,51,52]
4	940	polysaccharides (marker band for glycosidic linkage observed in starch)	[48,49,51,52,54]
5	1052	polysaccharides	[48,49,52,54]
6	1084	polysaccharides	[49]
7	1127	polysaccharides	[48,49,52]
8,9	1260, 1340	polysaccharides, lipids, fatty acids	[48,49,52,54]
10	1380	polysaccharides (deformation modes)	[48,49,51,54]
11	1457	polysaccharides, lipids, fatty acids	[48,49,54,55,56]
12	1600	polyphenols, flavonoids (stretching ring vibrations)	[51,57,58,59]
13	1660	amide I	[2,12,51]

**Table 2:** Conformations of S-S bridges [%] evaluated from Raman spectra of kernel endosperm of wasko.gl+ and wasko.gl− lines. The wavenumbers of the bands’ positions (cm^−1^), obtained by curve fitting spectra, are shown in the brackets.

Seed Lines	SSg-g-g [%]	SSt-g-g [%]	SSt-g-t [%]
wasko.gl+	18 (514)	46 (519, 524)	36 (531, 545)
wasko.gl−	15 (513)	39 (519, 525)	46 (533, 546)

**Table 3:** The estimated content [%] of the secondary structure of gliadin proteins isolated from flour obtained from kernels of wasko.gl− and wasko.gl+ wheat lines.

	wasko.gl−	wasko.gl+
β-sheet (1634–1640 cm^−1^)	19	12
α-helix (1649–1652 cm^−1^)	25	29
RC (1663 cm^−1^)	26	31
β-turn (1677–1678 cm^−1^)	27	25
aβ-sheet (1690–1692 cm^−1^)	3	3

## Data Availability

All data generated or analyzed during this study are included in this published article. Moreover, the datasets used and/or analyzed during the current study are available from the corresponding author on reasonable request.

## Supplementary Materials

The following are available online, Figure S1: The decomposition of the 500–550 cm^−1^ region obtained from the isolated gliadin proteins of wasko.gl+, panel (a), and wasko.gl−, panel (b). The experimental profiles are represented by dashed black lines and the calculated ones are represented by solid red lines. The calculated profiles in the panels were determined as the sums of the individual curve-fitted components typical for gauche–gauche–gauche (SS_g-g-g_) at 513(514)cm^−1^—blue line; for trans–gauche–gauche (SS_t-g-g_) in the range 519–525 cm^−1^—green line; and in the range 531–546 cm^−1^ for trans–gauche–trans (SS_t-g-t_) conformations—yellow line, respectively.

## Sample Availability

Not available.

## References

[1] Weselucha-Birczynska A., Labanowska M., Kurdziel M., Filek M. (2012). Resonance Raman and EPR spectroscopy studies of untreated spring wheat leaves. Vib. Spectrosc..

[2] Stawoska I., Staszak A.M., Ciereszko I., Oliwa J., Skoczowski A. (2020). Using isothermal calorimetry and FT-Raman spectroscopy for step-by-step monitoring of maize seed germination: Case study. J. Anal. Calorim..

[3] Saja D., Rys M., Stawoska I., Skoczowski A. (2016). Metabolic response of cornflower (*Centaurea cyanus* L.) exposed to tribenuron-methyl: One of the active substances of sulfonylurea herbicides. Acta Physiol. Plant..

[4] Nawrocka A., Krekora M., Niewiadomski Z., Szymanska-Chargot M., Krawecka A., Sobota A., Mis A. (2020). Effect of moisturizing pre-treatment of dietary fibre preparations on formation of gluten network during model dough mixing—A study with application of FT-IR and FT-Raman spectroscopy. LWT-Food Sci. Technol..

[5] Krombholz R., Lunter D. (2020). A New Method for In-Situ Skin Penetration Analysis by Confocal Raman Microscopy. Molecules.

[6] Saletnik A., Saletnik B., Puchalski C. (2021). Overview of Popular Techniques of Raman Spectroscopy and Their Potential in the Study of Plant Tissues. Molecules.

[7] Labanowska M., Weselucha-Birczynska A., Kurdziel M., Puch P. (2013). Thermal effects on the structure of cereal starches. EPR and Raman spectroscopy studies. Carbohydr. Polym..

[8] Filek M., Labanowska M., Kurdziel M., Weselucha-Birczynska A., Bednarska-Kozakiewicz E. (2016). Structural and biochemical response of chloroplasts in tolerant and sensitive barley genotypes to drought stress. J. Plant. Physiol..

[9] Moskal P., Weselucha-Birczynska A., Labanowska M., Filek M. (2019). Adaxial and abaxial pattern of Urtica dioica leaves analyzed by 2DCOS ATR-FTIR as a function of their growth time and impact of environmental pollution. Vib. Spectrosc..

[10] Kurdziel M., Dlubacz A., Weselucha-Birczynska A., Filek M., Labanowska M. (2015). Stable radicals and biochemical compounds in embryos and endosperm of wheat grains differentiating sensitive and tolerant genotypes—EPR and Raman studies. J. Plant. Physiol..

[11] Labanowska M., Kurdziel M., Filek M., Weselucha-Birczynska A. (2016). The impact of biochemical composition and nature of paramagnetic species in grains on stress tolerance of oat cultivars. J. Plant. Physiol..

[12] Stawoska I., Weselucha-Birczynska A., Regonesi M.E., Riva M., Tortora P., Stochel G. (2009). Interaction of selected divalent metal ions with human ataxin-3 Q36. J. Biol. Inorg. Chem..

[13] Peticolas W.L. (1975). Applications of Raman-spectroscopy to biological macromolecules. Biochimie.

[14] Maiti N.C., Apetri M.M., Zagorski M.G., Carey P.R., Anderson V.E. (2004). Raman spectroscopic characterization of secondary structure in natively unfolded proteins: Alpha-synuclein. J. Am. Chem. Soc..

[15] Lancelot E., Fontaine J., Grua-Priol J., Assaf A., Thouand G., Le-Bail A. (2021). Study of structural changes of gluten proteins during bread dough mixing by Raman spectroscopy. Food Chemistry.

[16] Sadat A., Joye I.J. (2020). Peak Fitting Applied to Fourier Transform Infrared and Raman Spectroscopic Analysis of Proteins. Appl. Sci..

[17] Tu A.T. (1982). Raman spectroscopy in biology: Principles and applications.

[18] Lefevre T., Rousseau M.E., Pezolet M. (2007). Protein secondary structure and orientation in silk as revealed by Raman spectromicroscopy. Biophys. J..

[19] Chi Z.H., Chen X.G., Holtz J.S.W., Asher S.A. (1998). UV resonance Raman-selective amide vibrational enhancement: Quantitative methodology for determining protein secondary structure. Biochemistry.

[20] De Gelder J., De Gussem K., Vandenabeele P., Moens L. (2007). Reference database of Raman spectra of biological molecules. J. Raman Spectrosc..

[21] Rygula A., Majzner K., Marzec K.M., Kaczor A., Pilarczyk M., Baranska M. (2013). Raman spectroscopy of proteins: A review. J. Raman Spectrosc..

[22] Ridgley D.M., Claunch E.C., Barone J.R. (2013). Characterization of Large Amyloid Fibers and Tapes with Fourier Transform Infrared (FT-IR) and Raman Spectroscopy. Appl. Spectrosc..

[23] Tuma R. (2005). Raman spectroscopy of proteins: From peptides to large assemblies. J. Raman Spectrosc..

[24] Sapone A., Bai J.C., Ciacci C., Dolinsek J., Green P.H.R., Hadjivassiliou M., Kaukinen K., Rostami K., Sanders D.S., Schumann M. (2012). Spectrum of gluten-related disorders: Consensus on new nomenclature and classification. BMC Med..

[25] Wrigley C., Békés F., Bushuk W., Wrigley C., Békés F., Bushuk W. (2006). Gluten: A balance of gliadin and glutenin. Gliadin and Glutenin. The Unique Balance of Wheat Quality.

[26] Shewry P.R., Tatham A.S., Halford N.G., Shewry P.R., Casey R. (1999). The prolamins of the Triticeae. Seed Proteins.

[27] Payne P., Holt L., Lister P. Gli-A3 and Gli-B3, two newly designated loci coding for omega-type gliadins and D subunits of glutenin. Proceedings of the 7th International Wheat Genetics Symposium.

[28] Payne P.I., Corfield K.G. (1979). Subunit composition of wheat glutenin proteins, isolated by gel-filtration in a dissociating medium. Planta.

[29] Wrigley C., Bietz J., Pomeranz Y. (1988). Proteins and amino acids. Wheat: Chemistry and Technology.

[30] Muller S., Wieser H. (1997). The location of disulphide bonds in monomeric gamma-type gliadins. J. Cereal Sci..

[31] Klosok K., Welc R., Fornal E., Nawrocka A. (2021). Effects of Physical and Chemical Factors on the Structure of Gluten, Gliadins and Glutenins as Studied with Spectroscopic Methods. Molecules.

[32] Tatham A.S., Schofield J.D. (1996). The structures of wheat proteins. Wheat Structure: Biochemistry and Functionality.

[33] Shewry P., Tatham A. (1997). Disulphide bonds in wheat gluten proteins. J. Cereal Sci..

[34] Minkiewicz P., Sokolowska J., Darewicz M. (2015). The occurrence of sequences identical with epitopes from the allergen Pen a 1.0102 among food and non-food proteins. Pol. J. Food Nutr. Sci..

[35] Mameri H., Brossard C., Gaudin J.C., Gohon Y., Paty E., Beaudouin E., Moneret-Vautrin D.A., Drouet M., Sole V., Wien F. (2015). Structural Basis of IgE Binding to alpha- and gamma-Gliadins: Contribution of Disulfide Bonds and Repetitive and Nonrepetitive Domains. J. Agric. Food Chem..

[36] Matsuo H., Morita E., Tatham A.S., Morimoto K., Horikawa T., Osuna H., Ikezawa Z., Kaneko S., Kohno K., Dekio S. (2004). Identification of the IgE-binding epitope in omega-5 gliadin, a major allergen in wheat-dependent exercise-induced anaphylaxis. J. Biol. Chem..

[37] Battais F., Mothes T., Moneret-Vautrin D.A., Pineau F., Kanny G., Popineau Y., Bodinier M., Denery-Papini S. (2005). Identification of IgE-binding epitopes on gliadins for patients with food allergy to wheat. Allergy.

[38] Rustgi S., Shewry P., Brouns F., Deleu L., Delcour J.A. (2019). Wheat Seed Proteins: Factor's Influencing Their Content, Composition, and Technological Properties, and Strategies to Reduce Adverse Reactions. Compr. Rev. Food Sci. Food Saf..

[39] Leszczynska J., Waga J., Lacka A., Wolska K., Majak I., Bartos A. (2013). The effect of enzymatic modification and genetic background on wheat gliadin immunological properties. Food Agric. Immunol..

[40] Barro F., Iehisa J.C.M., Gimenez M.J., Garcia-Molina M.D., Ozuna C.V., Comino I., Sousa C., Gil-Humanes J. (2016). Targeting of prolamins by RNAi in bread wheat: Effectiveness of seven silencing-fragment combinations for obtaining lines devoid of coeliac disease epitopes from highly immunogenic gliadins. Plant. Biotechnol. J..

[41] Waga J., Skoczowski A. (2014). Development and characteristics of ω-gliadin-free wheat genotypes. Euphytica.

[42] Skoczowski A., Obtulowicz K., Czarnobilska E., Dyga W., Mazur M., Stawoska I., Waga J. (2017). Antibody reactivity in patients with IgE-mediated wheat allergy to various subunits and fractions of gluten and non-gluten proteins from omega-gliadin-free wheat genotypes. Ann. Agric. Environ. Med..

[43] Cho S.W., Kang C.S., Kang T.G., Cho K.M., Park C.S. (2018). Influence of different nitrogen application on flour properties, gluten properties by HPLC and end-use quality of Korean wheat. J. Integr. Agric..

[44] Bietz J.A., Gooding K.M., Regnier F.E. (2002). HPLC of cereal endosperm storage proteins. HPLC of Biological Macromolecules, 2nd Edition, Revised and Expanded.

[45] Becker D., Folck A., Wieser H. (2006). Inhibierung der α-Gliadin-Genexpression in hexaploidem Brotweizen. Getreidetechnologie.

[46] Kumamarum T., Ogawa M., Satoh H., Okita T.W., Olsen O.A. (2007). Protein Body Biogenesis in Cereal Endosperms. Endosperm. Plant Cell Monographs (8).

[47] Fan D.M., Ma W.R., Wang L.Y., Huang J.L., Zhao J.X., Zhang H., Chen W. (2012). Determination of structural changes in microwaved rice starch using Fourier transform infrared and Raman spectroscopy. Starch-Starke.

[48] Kizil R., Irudayaraj J., Seetharaman K. (2002). Characterization of Irradiated Starches by Using FT-Raman and FTIR Spectroscopy. J. Agric. Food Chem..

[49] Liu Y.Q., Xu Y., Yan Y.Z., Hu D.D., Yang L.Z., Shen R.L. (2015). Application of Raman spectroscopy in structure analysis and crystallinity calculation of corn starch. Starch-Starke.

[50] Corbett E.C., Zichy V., Goral J., Passingham C. (1991). Fourier transform Raman studies of materials and compounds of biological importance—II. The effect of moisture on the molecular structure of the alpha and beta anomers of d-glucose. Spectrochim. Acta A.

[51] Schulz H., Baranska M. (2007). Identification and quantification of valuable plant substances by IR and Raman spectroscopy. Vib. Spectrosc..

[52] Wiercigroch E., Szafraniec E., Czamara K., Pacia M.Z., Majzner K., Kochan K., Kaczor A., Baranska M., Malek K. (2017). Raman and infrared spectroscopy of carbohydrates: A review. Spectrochim. Acta Pt. A-Mol. Biomol. Spectroc..

[53] Kacurakova M., Mathlouthi M. (1996). FTIR and laser-Raman spectra of oligosaccharides in water: Characterization of the glycosidic bond. Carbohydr. Res..

[54] Almeida M.R., Alves R.S., Nascimbem L., Stephani R., Poppi R.J., de Oliveira L.F.C. (2010). Determination of amylose content in starch using Raman spectroscopy and multivariate calibration analysis. Anal. Bioanal. Chem..

[55] Petrou M., Edwards H.G.M., Janaway R.C., Thompson G.B., Wilson A.S. (2009). Fourier-Transform Raman spectroscopic study of a Neolithic waterlogged wood assemblage. Anal. Bioanal Chem.

[56] Durmaz S., Ozgenc O., Boyaci I.H., Yildiz U.C., Erisir E. (2016). Examination of the chemical changes in spruce wood degraded by brown-rot fungi using FT-IR and FT-Raman spectroscopy. Vib. Spectrosc..

[57] Schrader B., Klump H.H., Schenzel K., Schulz H. (1999). Non-destructive NIR FT Raman analysis of plants. J. Mol. Struct..

[58] Vitek P., Novotna K., Hodanova P., Rapantova B., Klem K. (2017). Detection of herbicide effects on pigment composition and PSII photochemistry in Helianthus annuus by Raman spectroscopy and chlorophyll a fluorescence. Spectrochim. Acta Pt. A-Mol. Biomol. Spectroc..

[59] Heredia-Guerrero J.A., Benitez J.J., Dominguez E., Bayer I.S., Cingolani R., Athanassiou A., Heredia A. (2014). Infrared and Raman spectroscopic features of plant cuticles: A review. Front. Plant. Sci..

[60] Baranska M., Schulz H., Baranski R., Nothnagel T., Christensen L.P. (2005). In Situ Simultaneous Analysis of Polyacetylenes, Carotenoids and Polysaccharides in Carrot Roots. J. Agric. Food Chem..

[61] Yang L.Q., Zhang L.M. (2009). Chemical structural and chain conformational characterization of some bioactive polysaccharides isolated from natural sources. Carbohydr. Polym..

[62] Mahdad-Benzerdjeb A., Taleb-Mokhtari I.N., Sekkal-Rahal M. (2007). Normal coordinates analyses of disaccharides constituted by D-glucose, D-galactose and D-fructose units. Spectrochim. Acta Pt. A-Mol. Biomol. Spectroc..

[63] LiChan E.C.Y. (1996). The applications of Raman spectroscopy in food science. Trends Food Sci. Technol..

[64] Siamwiza M.N., Lord R.C., Chen M.C., Takamatsu T., Harada I., Matsuura H., Shimanouchi T. (1975). Interpretation of doublet at 850 and 830 cm-1 in Raman spectra of tyrosyl residues in proteins and certain model compounds. Biochemistry.

[65] Nawrocka A., Szymanska-Chargot M., Mis A., Ptaszynska A.A., Kowalski R., Wasko P., Gruszecki W.I. (2015). Influence of dietary fibre on gluten proteins structure—a study on model flour with application of FT-Raman spectroscopy. J. Raman Spectroc..

[66] Ferrer E.G., Gomez A.V., Anon M.C., Puppo M.C. (2011). Structural changes in gluten protein structure after addition of emulsifier. A Raman spectroscopy study. Spectrochim. Acta Pt. A-Mol. Biomol. Spectroc..

[67] Meng G.T., Ma C.Y., Phillips D.L. (2003). Raman spectroscopic study of globulin from Phaseolus angularis (red bean). Food Chem..

[68] Linlaud N., Ferrer E., Puppo M.C., Ferrero C. (2011). Hydrocolloid Interaction with water, protein, and starch in wheat dough. J. Agric. Food Chem..

[69] Nawrocka A., Mis A., Szymanska-Chargot M. (2016). Characteristics of relationships between structure of gluten proteins and dough rheology—influence of dietary fibres studied by FT-raman spectroscopy. Food Biophys..

[70] Zhou Y., Zhao D., Foster T.J., Liu Y.X., Wang Y., Nirasawa S., Tatsumi E., Cheng Y.Q. (2014). Konjac glucomannan-induced changes in thiol/disulphide exchange and gluten conformation upon dough mixing. Food Chem..

[71] Nawrocka A., Szymanska-Chargot M., Mis A., Kowalski R., Gruszecki W.I. (2016). Raman studies of gluten proteins aggregation induced by dietary fibres. Food Chem..

[72] Tatham A.S., Shewry P.R. (1985). The conformation of wheat gluten proteins—The secondary structures and thermal stabilities of alpha-gliadins, beta-gliadins, gamma-gliadins and omega-gliadins. J. Cereal Sci..

[73] Lindsay M.P., Skerritt J.H. (1999). The glutenin macropolymer of wheat flour doughs: Structure-function perspectives. Trends Food Sci. Technol..

[74] Feng J., ZHANG S., ZHANG Y., Jinshui W. (2018). Correlation of gluten molecular conformation with dough viscoelastic properties during storage. Grain Oil Sci. Technol..

[75] Peng H., Li B., Tian J. (2019). Impact of Punicalagin on the Physicochemical and Structural Properties of Wheat Flour Dough. Foods.

[76] Bushuk W., Zillman R.R. (1978). Wheat cultivar identification by gliadin electrophoregrams. 1. Apparatus, method and nomenclature. Can. J. Plant. Sci..

[77] Metakovsky E., Novoselskaya A. (1991). Gliadin allele identification in common wheat I: Methodological aspects of the analysis of gliadin patterns by one-dimensional polyacrylamide gel electrophoresis. J. Genet. Breed..

[78] Laemmli U.K. (1970). Cleavage of structural proteins during the assembly of the head of bacteriophage T4. Nature.

[79] James L.C., Roversi P., Tawfik D.S. (2003). Antibody multispecificity mediated by conformational diversity. Science.

[80] Varjonen E., Vainio E., Kalimo K. (2000). Antigliadin IgE—indicator of wheat allergy in atopic dermatitis. Allergy.

[81] Metsalu T., Vilo J. (2015). ClustVis: A web tool for visualizing clustering of multivariate data using Principal Component Analysis and heatmap. Nucleic Acids Res..

