# Supplementary material for: FT-Raman Spectroscopy as a Tool to Study the Secondary Structures of Wheat Gliadin Proteins
Source: Molecules. 2021 Sep 4;26(17):5388. doi: 10.3390/molecules26175388 (PMC8434250; doi:10.3390/molecules26175388)
Supplement: Supplementary file 1 [file molecules-26-05388-s001.zip › molecules-1342017-supplementary.pdf]

molecules

MDPI

Article

FT-Raman Spectroscopy as a Tool to Study the Secondary Structures of Wheat Gliadin Proteins

Iwona Stawoska [1]*, Aleksandra Wesełucha-Birczyńska [2], Andrzej Skoczowski [1,3], Michał Dziurka [3], Jacek Waga [4]*

[1] Institute of Biology, Pedagogical University of Krakow, Podchorążych 2, 30-084 Krakow, Poland; andrzej.skoczowski@up.krakow.pl
[2] Faculty of Chemistry, Jagiellonian University, Gronostajowa 2, 30-387 Kraków, Poland; birczyns@chemia.uj.edu.pl
[3] The Franciszek Górski Institute of Plant Physiology, Polish Academy of Sciences, Niezapominajek 21, 30-239 Krakow, Poland; michal.dziurka@gmail.com
[4] Department of Physiology, Plant Breeding and Seed Science, University of Agriculture, Podłużna 3, 30-239 Kraków, Poland
* Correspondence: iwona.stawoska@up.krakow.pl (I.S.); jacek.waga@urk.edu.pl (J.W.)

Citation: Stawoska, I.; Wesełucha-Birczyńska, A.; Skoczowski, A.; Dziurka, M.; Waga, J.; FT-Raman Spectroscopy as a Tool to Study the Secondary Structures of Wheat Gliadin Proteins. Molecules **2021**, 26, 5388. https://doi.org/10.3390/molecules26175388

Academic Editors: Paola Taddei and Michele Di Foggia

Received: 30 July 2021
Accepted: 2 September 2021
Published: 4 September 2021

Publisher's Note: MDPI stays neutral with regard to jurisdictional claims in published maps and institu-tional affiliations.

Copyright: © 2021 by the authors. Submitted for possible open access publication under the terms and conditions of the Creative Commons Attribution (CC BY) license (https://creativecommons.org/licenses/by/4.0/).

Supplementary Materials:

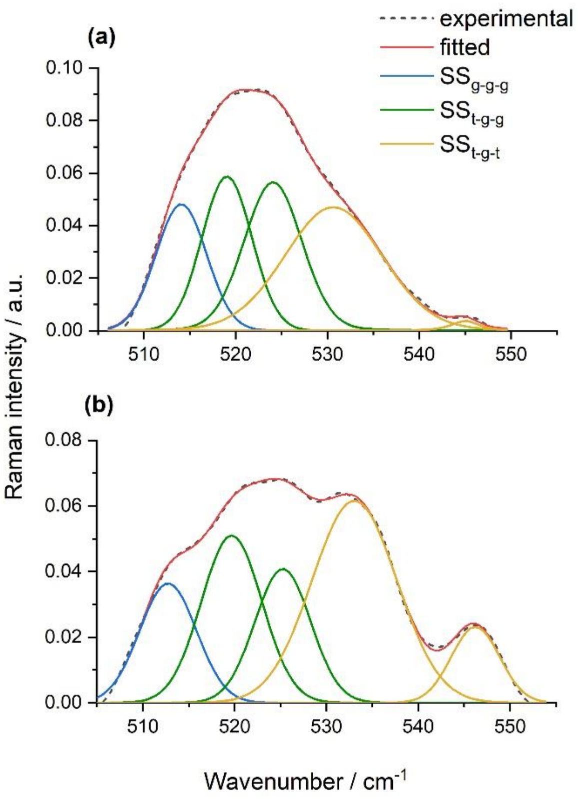

Figure S1. The decomposition of the 500–550 cm^{-1} region obtained from the isolated gliadin proteins of wasko.gl+, panel (a), and wasko.gl–, panel (b). The experimental profiles are represented by dashed black lines and the calculated ones are represented by solid red lines. The calculated profiles in the panels were determined as the sums of the individual curve-fitted components typical for gauche–gauche–gauche (SS_{g-g-g}) at 513(514)cm^{-1}—blue line; for trans–gauche–gauche (SS_{t-g-g}) in the

range 519–525 cm^{-1}—green line; and in the range 531–546 cm^{-1} for trans–gauche–trans ($SS_{t\text{-}g\text{-}t}$) conformations—yellow line, respectively.
